# Changes in Diffusion MRI-Based White Matter Metrics After a Dance Intervention in Individuals with Substance Use Disorders—A Pilot Study

**DOI:** 10.3390/brainsci16101050

**Published:** 2026-09-29

**Authors:** Anna Kannonier, Sandria Stornig, Karl Koschutnig, Human-Friedrich Unterrainer, Patrick Klepits, Andreas Fink

**Affiliations:** 1 Department of Psychology, University of Graz, 8010 Graz, Austria; s.stornig@edu.uni-graz.at (S.S.); patrick.klepits@edu.uni-graz.at (P.K.); andreas.fink@uni-graz.at (A.F.); 2 Department of Human Movement Sciences, University of Graz, 8010 Graz, Austria; 3 MRI-Lab Graz, University of Graz, 8010 Graz, Austria; karl.koschutnig@uni-graz.at; 4 Faculty of Psychotherapy Science, Sigmund Freud University Vienna, 1020 Vienna, Austria; human.unterrainer@sfu.ac.at; 5 Department of Religious Studies, University of Vienna, 1010 Vienna, Austria; 6 Division of Psychiatry and Psychotherapeutic Medicine, Medical University of Graz, 8036 Graz, Austria; 7 Center for Integrative Addiction Research (CIAR), Grüner Kreis Organization, 1110 Vienna, Austria

**Keywords:** dance, substance use disorders, fractional anisotropy (FA), quantitative anisotropy (QA)

## Abstract

**Highlights:**

**What are the main findings?**

- Dancing may induce white matter reorganization in individuals with substance use disorders.
- White matter changes were particularly prominent in the cerebellum.

**What are the implications of the main findings?**

- Dance-based interventions may provide a promising complementary approach to supporting recovery in substance use disorders.
- Understanding neural adaptations associated with (motor) learning may help tailor movement-based interventions to clinical populations.

**Abstract:**

**Objectives**: The present study examined the effects of a three-week dance intervention on diffusion MRI-based white matter metrics in a sample of individuals diagnosed with substance use disorder (SUD; F19.2). **Methods**: The intervention was carried out in an inpatient rehabilitation facility twice a week. Participants were partly randomized into two groups, one intervention group and one treatment-as-usual group, which did not differ in terms of substitution or other psychiatric medications. Both groups underwent an MRI assessment at baseline and after the intervention, using diffusion-weighted imaging. **Results**: Analysis of MRI diffusion data revealed significant effects in the intervention group relative to the treatment-as-usual group, particularly within white matter networks associated with sensorimotor functions, with the most pronounced changes in the cerebellum. **Conclusions**: These findings suggest that learning a new motor skill may induce white matter changes in the brain in individuals with SUDs.

## 1. Introduction

In 2019, 2.6 million deaths were caused by alcohol consumption and almost 600,000 deaths resulted from the use of psychoactive drugs [1]. The consequences of drug consumption and substance use disorders lead to a significant health, social, and economic burden, making it necessary to implement effective prevention and treatment interventions [1].

From a neurobiological perspective, regular drug consumption can have severe impacts on the human brain [2], with major pathways of white matter appearing to be particularly affected [3,4]. Chronic intake of alcohol, opiates and cocaine, for example, is generally associated with a lower coherence of white matter, often found in the corpus callosum, as well as the superior and inferior longitudinal fasciculus [3]. Similar findings have been shown in people with SUD, who consume more than one substance, revealing impairments in white matter integrity, especially in the corticospinal tract and the inferior longitudinal fasciculi [4].

However, it is unclear to what extent such alterations are permanent [3]. There is limited research on how different treatments, including pharmacological and behavioral treatments, could counteract the adverse neurobiological effects of long-term drug use. Especially for behavioral interventions, measuring and identifying the underlying neurobiological mechanisms of intervention-related effects is a complex endeavor.

An important factor in the treatment of SUDs is the integration of medical, psychological, social, and recreational aspects to support people in all aspects of their lives. Although recreational and leisure activities are often implemented in SUD rehabilitation programs, there is a lack of evidence-based studies supporting their effectiveness. In this context, a promising approach to supporting SUD patients in their recovery is physical exercise. Physical exercise has been shown to reduce anxiety and depression levels, decrease cravings, improve psychosocial well-being, and contribute to reductions in substance use [5,6].

Based on neuroscientific research, there are numerous studies that show the beneficial effects of physical exercise on functional and structural characteristics of the brain [7,8,9]. Notably, such changes have been observed after relatively short training periods of 2–3 weeks. Evidence from intervention studies demonstrates volumetric increases in several subfields of the hippocampus and alterations in white matter integrity following running, unicycling, and slackline training [10,11,12].

These findings could be especially relevant for specific target groups, such as clinical populations or elderly people. Especially exercises involving motor and visuospatial coordination are believed to be particularly responsive to higher cognitive functions [13]. Dance, for example, is a type of movement that involves a variety of cognitive and affective functions, including the coordination of body movement, timing, memory, visuospatial perception, and emotional expression. Studies have shown that regular participation in dancing can promote structural changes in the brain [14,15], improve related aspects of mental and physical health, and increase levels of neurotrophic factors [15]. In healthy seniors, for example, regular dancing has been found to increase hippocampal volume and white matter integrity in the corpus callosum [16].

In clinical settings, evidence suggests dancing to be a promising approach to improving motor and cognitive functions, overall functionality, and alleviating mental symptoms in patients with neurological disorders, including patients with Parkinson’s disease [17,18] and Alzheimer’s disease [19].

In the context of substance use disorders, dancing could be a promising therapy adjunct in the recovery process. It not only incorporates physical exercise and cognitive demands, but also emphasizes body awareness and perception. This may be particularly beneficial for individuals with SUDs, as regular substance consumption can decrease bodily reactivity and lead to a reduced interoceptive perception [20]. In view of the adverse effects drug use can have on the brain, we are particularly interested in exploring brain plasticity and the effects of dancing from a neuroscientific perspective.

Hence, the present pilot study examined the effects of a dance intervention on diffusion MRI–based metrics—quantitative anisotropy (QA) and fractional anisotropy (FA)—in a sample of participants with a history of substance use disorders, using magnetic resonance imaging (MRI). We expected intervention-related changes in FA and QA, specifically in pathways related to motor and cognitive functions.

## 2. Method

### 2.1. Participants

**Figure 1:**
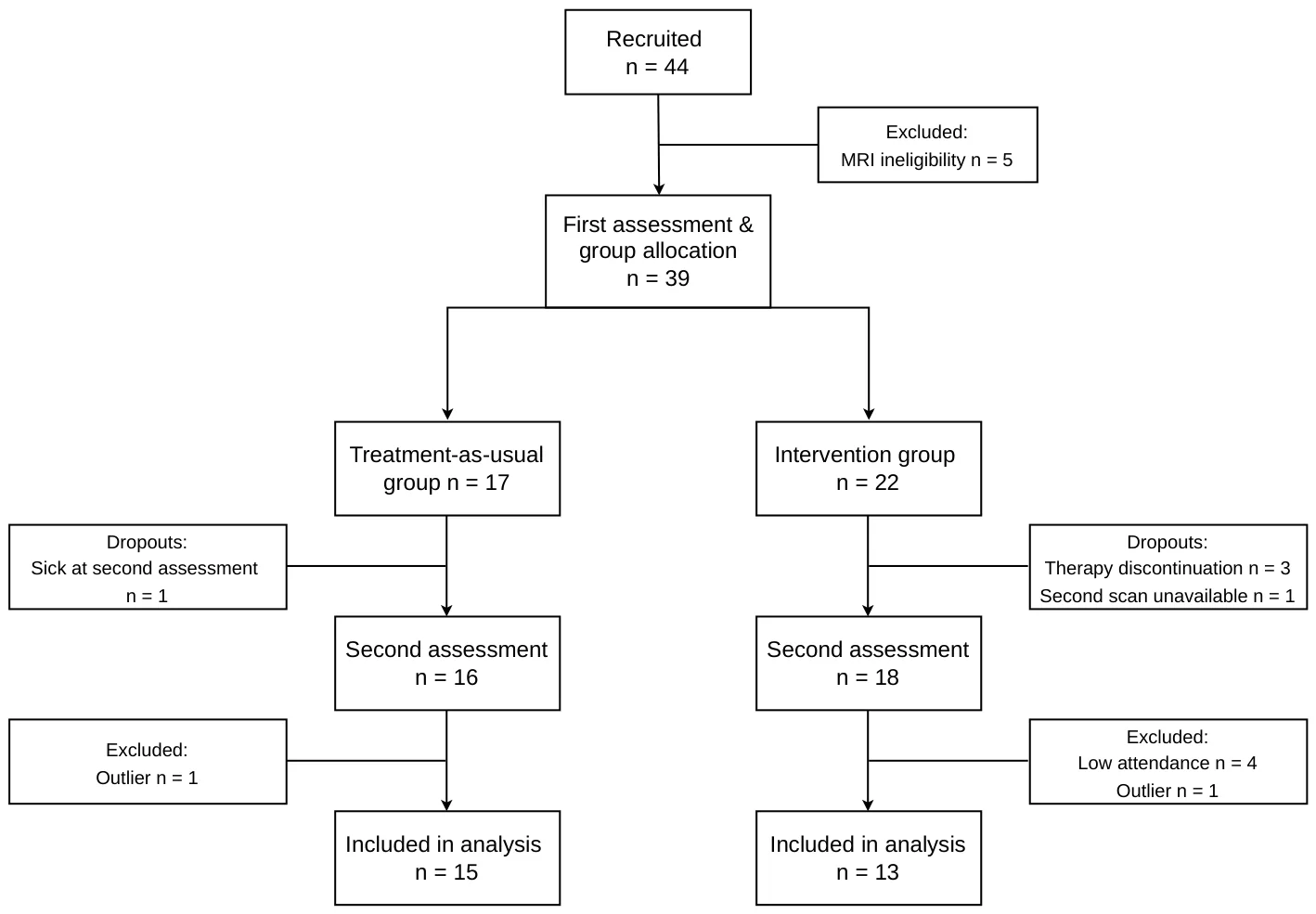
Participant flow diagram.

A sample of 44 participants, diagnosed with “mental and behavioral disorders due to multiple drug use and the use of other psychoactive substances: dependence syndrom” (ICD-10: F19.2), was recruited. At the time of this study, all participants were in long-term treatment (6–18 months) for SUD in an inpatient therapeutic facility (Grüner Kreis, Austria; https://gruenerkreis.at/en; accessed on 27 August 2026). Before the first MRI session, participants were screened for MRI eligibility and right-handedness. Of the 44 participants, 28 were included in the final MRI analysis. Dropouts occurred due to ineligibility or inability to complete an MRI scan, discontinuation of therapy, low attendance at intervention sessions (at least 3 out of 6 sessions) and illness. The flow diagram in Figure 1 shows the recruitment process and the number of dropouts during the study period. The final sample consisted of 4 women and 24 men with an age range between 19 and 51 years (*M* = 30.61, SD = 8.52). A total of 16 of them had comorbid diagnoses (other than F19.2), including depressive disorders (*n* = 5), hyperkinetic disorders (*n* = 5), post-traumatic stress and adjustment disorders (*n* = 3), mixed personality disorders (*n* = 3), schizophrenia (*n* = 2), unspecified bipolar affective disorder (*n* = 2), mixed anxiety and depressive disorder (*n* = 1), eating disorder (*n* = 1), epilepsy (*n* = 1) and HIV (*n* = 1). Eight were taking substitute medication and 22 were taking other psychiatric medications. Substances consumed before treatment included alcohol, opiates, and a range of illicit drugs. A detailed overview of the final sample is shown in Table 1. This study was approved by the local authorized ethics committee of the University of Graz.

### 2.2. Study Design and Procedure

**Figure 2:**
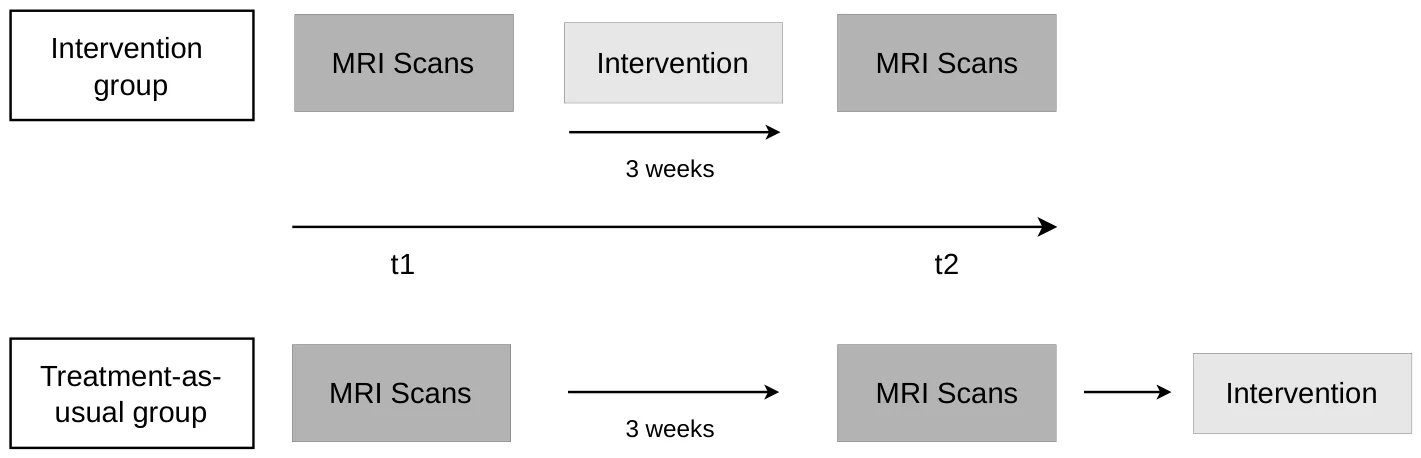
Study Design. Both groups underwent MRI assessment at baseline (t1) and post-intervention (t2). The intervention group received a three-week dance intervention, while the treatment-as-usual group continued their regular rehabilitation routine. Participants in the treatment-as-usual group were offered dance training after their post-test.

A mixed within- and between-subjects, quasi-experimental design was used. Participants were assigned to one of two groups, an intervention group and a treatment-as-usual group. The intervention group received structured dance training, embedded in their daily therapy structure in the rehabilitation facility, while the treatment-as-usual group continued with their regular rehabilitation routine. Dance training was organized over a three-week period, involving two sessions per week, each session lasting 90 min. Both groups underwent a magnetic resonance assessment at baseline and post-intervention, using diffusion-weighted imaging to measure white matter integrity. Participants in the treatment-as-usual group were offered dance training after their post-test. An overview of the study design can be found in Figure 2.

The sample was the result of two rounds of data collection. In the first round, participants were randomly assigned to one of the two groups. Based on the higher dropout rate observed in the intervention group during the first round, the intervention group was oversampled in the second round to ensure an adequate sample size in both groups. For ethical reasons, one participant was reassigned from the treatment-as-usual group to the intervention group before the start of the intervention. This participant would otherwise not have been able to attend the dance training offered to the treatment-as-usual group after the post-test. These changes in group assignment resulted in an overall higher proportion of participants being allocated to the intervention group. This decision was based on the experience during the first round of data collection, in which participants often had concurrent therapies or scheduled activities overlapping with the dance sessions. Additionally, intrinsic motivation and high symptom load may have contributed to higher dropout rates in the intervention group.

### 2.3. Dance Intervention

Dance sessions were held in a room within the facility that was easily accessible to the participants. The general structure of the class comprised the following components: (1) warm-up and dance technique, (2) partner or group work, (3) creative dance activities, (4) choreography practice and (5) cool-down. The content incorporated elements of contemporary dance, street dance, and classical ballet, including technical aspects and artistic exploration. Sessions were led by two female instructors using a co-teaching model: one guided the exercises while the other participated alongside the group or observed. The roles were rotated three times per session.

The main goals were to support the participants in learning basic dance techniques, engage them in movement activities that involve touch and collaboration, and apply the learned material to choreographic tasks. The classes aimed to create a safe and joyful environment, promote body awareness, and expand the movement repertoire of each participant. Various teaching methods were used, including verbal guidance, mirroring, demonstration, and alternation between internal and external attentional focus. At the beginning of each session involving physical contact, participants were asked to close their eyes and indicate whether they consented to touch that day.

Details of the intervention are described in the Supplementary Materials.

### 2.4. MRI Data Acquisition

At the MRI-Lab Graz in Austria, magnetic resonance imaging (MRI) data were acquired on a 3.0 T Siemens MAGNETOM Vida system (Siemens Healthineers, Erlangen, Germany) equipped with a 64-channel head coil. Multishell diffusion-weighted data were obtained using a two-dimensional spin-echo, single-shot, multiband echo-planar imaging (EPI) sequence comprising 60 slices, with a repetition time (TR) of 3500 ms, an isotropic voxel size of 2 mm, and an acceleration factor of 4. To optimize acquisition efficiency for each shell, the echo time (TE) was minimized accordingly.

Two diffusion shells with increasing numbers of diffusion-encoding directions were acquired (b value/number of directions/TE = 1000 s/mm^2^/20/92 ms, 3000 s/mm^2^/64/106 ms). For each shell, two initial non-diffusion-weighted volumes (b = 0 s/mm^2^) were collected with a TE matched to that of the corresponding shell. To enable susceptibility-induced distortion correction, two additional b0 images per shell were acquired with reversed phase-encoding polarity (posterior–anterior).

Furthermore, a high-resolution, submillimeter three-dimensional MPRAGE scan was acquired (224 slabs; TR/TE/inversion time = 2970/2.06/1000 ms; 0.9 mm isotropic voxels; flip angle = 8°).

### 2.5. Data Preprocessing

Preprocessing was conducted using QSIPrep (version 22.1; [21]), which is implemented on top of Nipype 1.9.1 [22]. The T1-weighted anatomical image was aligned to the anterior commissure–posterior commissure (AC–PC) plane, nonlinearly registered to the MNI152NLin2009cAsym template using the SyN algorithm, and subjected to brain extraction with SynthStrip. Tissue segmentation was performed with SynthSeg (FreeSurfer v7.3.1).

For the diffusion-weighted data, all volumes with b-values < 100 s/mm^2^ were treated as b = 0 images. Marchenko–Pastur principal component analysis (MP-PCA) denoising [23] and N4 bias field correction [24] were carried out using MRtrix3. Head motion and eddy current-induced distortions were corrected with FSL’s eddy [25]. Susceptibility-induced distortions were corrected with TOPUP [26] using pairs of b = 0 images acquired with reversed phase-encoding directions. Shell alignment and outlier detection/correction procedures were additionally applied. Confounding regressors, including frame-wise displacement, were derived for subsequent analyses. The final diffusion-weighted imaging (DWI) datasets were resampled into AC–PC space with an isotropic voxel size of 2 mm.

### 2.6. Measures of White Matter Reorganization

Following preprocessing, the diffusion data were reconstructed in DSI Studio (Hou Version; 27 March 2026, https://dsi-studio.labsolver.org/ (accessed on 27 August 2026)) using q-space diffeomorphic reconstruction [27], which nonlinearly normalizes the data into ICBM-152 template space. A diffusion sampling length ratio of 1.25 was used. The output resolution matched the template’s native voxel size. Quantitative anisotropy (QA) was derived for each fiber population, and fractional anisotropy (FA) was calculated from the diffusion tensor fitted to the b ≤ 1750 s/mm^2^ shell. The reconstructed FIB files were aggregated into a group-level connectometry database.

Our primary diffusion metric is quantitative anisotropy (QA). It yields a separate value for each distinct fiber population within a voxel, thereby providing a highly specific measure, particularly in regions containing crossing or branching fiber pathways [28,29]. Consequently, QA is considered more sensitive to subtle microstructural variations and more reflective of axonal loss than many conventional diffusion metrics [29]. In line with this, multiple studies have documented QA reductions across various clinical populations [30].

In addition to QA, we also examined fractional anisotropy (FA), the most commonly employed index of white matter integrity, to enhance comparability with the existing literature. In contrast to QA, which can be estimated at the level of individual fiber populations, FA provides a voxel-wise summary of diffusion anisotropy by aggregating the contributions of all fiber populations within a voxel [28,29].

### 2.7. Statistical Analysis

Data were processed using DSI Studio (Version 27 March 2026). This software implements connectometry-based correlational tractography to identify white matter segments associated with a given study variable [31].

Before the analysis, the quality parameters of the scans were checked. Two subjects were marked as outliers (due to lower neighboring correlation values) and thus excluded from the subsequent analysis.

To investigate the effects of the dance intervention, “group” was chosen as a study variable. Longitudinal alterations in QA and FA were computed by subtracting values at scan 1 (baseline) from those at scan 2 (post-intervention). A non-parametric Spearman partial correlation analysis was conducted, controlling for age and sex via a multiple regression model. A deterministic fiber tracking algorithm [32] was applied using effect size thresholds of 0.25 (T threshold = 1.4), 0.30 (T threshold = 1.7), 0.35 (T threshold = 2.0), and 0.40 (T threshold = 2.4). The minimum fiber length was set to 20 voxels (corresponding to 40 mm). Whole-brain tractography was performed, and resultant streamlines were refined using topology-informed pruning [33] with 16 iterations. To control the false discovery rate (FDR) at 0.05, 10,000 randomized permutations were performed.

Subsequent processing of the tractography output was carried out in DSI Studio using the “Recognize and Cluster” function. To enhance methodological transparency, results are reported across multiple effect size thresholds (from ρ = 0.25 to ρ = 0.40 in increments of 0.05), thereby demonstrating how the observed effects vary as the analysis criteria are made progressively more conservative.

Participants’ substance-use history, medication, and comorbid diagnoses were not included as confounders in the diffusion analysis. However, we compared the groups regarding the number of individuals with comorbid diagnoses, substitution treatment, psychiatric medication (non-subst.), alcohol use, opiate use, and illicit drug use. These comparisons did not reveal any significant group differences.

## 3. Results

### 3.1. QA

The connectometry analysis revealed that longitudinal changes in QA were significantly associated with group, up to an effect size of ρ = 0.40 (FDR ≤ 0.05). Specifically, the intervention group, relative to the treatment-as-usual group, showed decreases in the bilateral cerebellum and the cerebellar vermis, brain regions that are related to movement coordination, balance, and motor learning [34,35]. These decreases were most pronounced in the right cerebellum. A detailed overview of the results is illustrated in Table 2. Volume was chosen as a shape description, as it captures a quantified measurement of structural shape characteristics of fiber pathways [36].

### 3.2. FA

Furthermore, the analysis revealed significant effects on longitudinal changes in FA up to an effect size of ρ = 0.40 (FDR ≤ 0.05) in the intervention group relative to the treatment-as-usual group. These effects were shown across widespread neural networks associated with motor and cognitive functions, including increases in parts of the corpus callosum, the left inferior longitudinal fasciculus, and the left corticostriatal tract, as well as decreases in the bilateral cerebellum, the right cingulum, and the body of the corpus callosum. A detailed overview of the results is illustrated in Table 3.

The significant findings in QA and FA are visually summarized in Table 4.

### 3.3. Baseline Differences

Given the heterogeneity of the sample, we additionally examined baseline differences between the two groups (effect size threshold: ρ = 0.30). This analysis revealed multiple group differences across several fiber tracts (see Supplementary Materials). Notably, the primary fiber tracts that show differences in longitudinal change between the groups, such as those in the cerebellum, were not evident in the baseline comparisons. Additionally, by analyzing within-subject change scores of white matter metrics, we place the emphasis on training-related adaptations. However, the potential influence of baseline group differences cannot be fully excluded, as their diffusion values have not been specifically accounted for in the longitudinal analysis.

## 4. Discussion

To the very best of our knowledge, this is the first study to provide preliminary evidence of early changes in diffusion-based white matter metrics following a dance-based intervention in people recovering from substance use disorder. Whole-brain diffusion analyses revealed significant effects in white matter tracts supporting motor and cognitive functions [35,37]. These changes were particularly evident in the bilateral cerebellum, parts of the corpus callosum, and the bilateral cingulum.

Learning a new motor skill, such as dance, requires the coordination of complex movements, adaptation to new demands, and the interaction of motor and sensory information. The cerebellum plays an essential role in these processes, as it is primarily involved in motor coordination and sensorimotor integration [38,39], and is fundamental for early motor learning [40]. Significant changes were also found in the cerebellar vermis, which is crucial for postural control and maintaining balance by processing sensory input from the environment to generate motor commands and coordinate movement [34].

As suggested by our findings, QA primarily showed decreases in white matter fibers, whereas FA displayed bidirectional changes across several regions of the brain. Consistent with the decreases in QA, the most prominent decreases in FA were observed in the cerebellum. In addition, declines were found in parts of the cingulum, an important pathway for emotional processing [41].

Moreover, our results revealed increases in FA in parts of the corpus callosum (e.g., body, forceps major) whose key function is the integration and coordination of inter-hemispheric information [42,43]. Similarly, Rehfeld et al. [16] found that dancing increases white matter integrity in the splenium of the corpus callosum, where the forceps major originates. Additional increases could be found in the corticostriatal tracts, which are closely related to motor functions [44]. These pathways support motor learning, planning, and execution processes [44] that play a critical role in dancing.

The observed findings may be particularly relevant for SUDs. Research on chronic use of cocaine, for example, has found that cocaine dependence negatively affects fine motor control and motor coordination [45], highlighting the potential relevance of dance and other movement-based interventions as complementary components of functional rehabilitation in SUDs. Given the exploratory nature of the present study, further research is necessary to investigate whether such changes in white matter are associated with clinically meaningful alterations in motor function.

Notably, the longitudinal changes in the cerebellum manifested as decreases in the intervention group relative to the treatment-as-usual group. This finding was observed for both white matter metrics (FA and QA) and remained evident at effect sizes up to ρ = 0.40. Such decreases do not necessarily reflect neural degeneration. Potential biological mechanisms cannot be directly derived from the observed changes, but there are several interesting mechanisms that have been proposed in association with decreases in white matter metrics [46]. For example, such decreases may reflect the expansion of axon diameters or the emergence of new fiber populations within regions of crossing fibers [46]. Furthermore, it is plausible that learning requires neural adjustment and reorganization within the brain, which may follow a non-linear process. Thus, the observed changes may indicate complex remodeling processes that cannot be simply explained by increases or decreases in measures of white matter integrity. This is not a novel finding, but has also been reported in other physical activity domains, for example after learning to walk on a slackline [12] or after learning to ride a unicycle [11]. However, the underlying cellular mechanisms cannot be established from diffusion-derived metrics, such as FA and QA, alone.

It is important to note that the observed effects may be temporary. For example, Thomas et al. [47] reported an increase in hippocampal volume after 6 weeks of aerobic exercise and conducted a follow-up scan after an additional 6-week period without training. They found that the intervention-induced changes had returned to baseline levels, suggesting that continuous engagement in physical activity is necessary to maintain these structural brain changes. However, other studies indicate that training-related adaptations can persist for several weeks without further engagement in the activity [12,48]. In particular, Scholz et al. [48] found that FA increases induced by juggling were still present after 4 weeks without practice, showing only a slight but non-significant reduction while remaining above baseline levels. These findings suggest that intervention-induced changes in the brain may be retained to some extent even without continued training, although their magnitude likely decreases over time.

Importantly, the treatment-as-usual group in the present study did not receive an activity-matched intervention. Thus, the multidimensional nature of the intervention should be considered when interpreting the findings. The dance sessions involved not only physical activity and motor learning, but also social interaction, music, play, and other non-specific elements, such as attention or enjoyment. Therefore, the observed findings may reflect the combined effects of these different components, making it difficult to distinguish the potential contribution of individual factors. Another factor to consider is that all participants were undergoing inpatient rehabilitation during the study period, which included various therapies and medications. In addition, the use of multiple drugs and potential drug interactions can have various neurobiological effects on the brain. This should be considered in the interpretation of the observed changes in this heterogeneous sample.

Nevertheless, the findings may illustrate the multimodal processes involved in dance, suggesting that learning such a highly demanding motor skill may be associated with early changes in diffusion-based white matter metrics. As dancing has already been found to support treatment outcomes in specific clinical populations [17,19], this pilot study provides a basis for further investigations of dance-based interventions across a wider range of clinical settings. Understanding the neurobiological mechanisms underlying these effects can help design and choose exercise- and movement-based interventions tailored to the needs of specific populations. In this context, dance is a very promising tool, as it can be designed and modified in terms of physical intensity, style, cognitive demands, and social dimensions.

Our findings provide preliminary evidence that dancing may contribute to the reorganization of white matter fiber tracts supporting motor learning in a clinical population. To better understand the potential clinical implications of these findings, future investigations are needed to examine the effects of dance- or movement-based interventions on clinical recovery outcomes.

## 5. Limitations

Among the most important limitations of the present study is the small sample size, as the comparatively high dropout rate resulted in a fairly low number of participants. The dropout from inpatient SUD treatments remains a constant challenge, as intrinsic motivation and high symptom load play an important role in this context. Because the intervention group was oversampled for this reason, the sample was only partly randomized. Thus, potential selection effects cannot be completely excluded. In addition, baseline differences in diffusion values between the groups were not specifically accounted for in the analysis, which should be considered in the interpretation of the findings. Another noteworthy limitation is the absence of a non-clinical control group. This may limit the interpretation of whether the observed effects are solely driven by the dance intervention or also by other treatment factors. Additionally, the treatment-as-usual group did not participate in a comparable activity during the intervention period. Therefore, factors such as social interaction, social support, or enjoyment cannot be fully excluded as potential non-specific intervention effects. However, it also appears noteworthy that the findings were most pronounced in specific networks that support processes involved in dancing (particularly the cerebellum or the corpus callosum). This specificity makes it less likely that the observed brain changes are due to other activities or aspects outside the intervention. Lastly, the intervention period was relatively short, which may limit the degree to which clinically meaningful changes can be observed. Furthermore, it remains unclear whether the findings of this study reflect temporary or longer-lasting changes. To address this issue, future studies should include a follow-up brain scan over an extended time period in order to assess the stability of these changes.

## Figures and Tables

**Table 1:** Overview of the final sample included in the MRI analyses.

	Treatment-as-Usual Group	Intervention Group
Sex		
Male	13	11
Female	2	2
Age		
Range	20–51	19–41
Mean (SD)	31.13 (10.18)	30.00 (6.46)
Comorbid Diagnoses	9	7
Substitution	4	4
Psychiatric medication (non-subst.)	13	9
Substances Consumed		
Alcohol	11	11
Opiates	9	10
Illicit Drugs	13	12

**Table 2:** Significant longitudinal changes (scan 2–scan 1) in QA in the intervention group relative to the treatment-as-usual group, volume [mm^3^] (FDR ≤ 0.05).

Effect Size	QA ↑	QA ↓
0.30	—	R Cerebellum, 3972 L Cerebellum, 1639 Cerebellar Vermis, 538
0.35	—	R Cerebellum, 1980
0.40	—	R Cerebellum, 375

Note: QA = quantitative anisotropy; ↑ increase; ↓ decrease. Volume [mm^3^] refers to the total spatial volume of the resulting tracts, calculated as the number of voxels multiplied by voxel size. The effect size threshold corresponds to the Spearman correlation coefficient (ρ).

**Table 3:** Significant longitudinal changes (scan 2–scan 1) in FA in the intervention group relative to the treatment-as-usual group, volume [mm^3^] (FDR ≤ 0.05).

Effect Size	FA ↑	FA ↓
0.30	CC Body, 4265 Forceps Major, 2254 L ILF, 1073 Tapetum, 935 L/R IFOF, 821/129 L Cerebellum, 733 L/R Corticostriatal Tract, 639/80 L/R Optic Radiation, 551/56 Anterior Commissure, 421 L Corticopontine Tract, 48	R/L Cerebellum, 5110/4104 CC Body, 1502 L/R Cingulum, 876/698 Middle Cerebellar Peduncle, 705 R/L Parolfactory Cingulum, 354/295 Tapetum, 323 Vermis, 175 L Parahippocampal Cingulum, 85 R/L SLF, 62/62
0.35	CC Body, 1011 Forceps Major, 377 Tapetum, 255 L ILF, 252 L Corticostriatal Tract, 225 L IFOF, 156	R/L Cerebellum, 2163/657 L/R Cingulum, 616/418 CC Body, 512 R/L Parolfactory Cingulum, 253/108 L Parahippocampal Cingulum, 60 L SLF, 51 Vermis, 53
0.40	—	R Cerebellum, 1109 L Cingulum, 200

Note: FA = fractional anisotropy; ↑ increase; ↓ decrease. Volume [mm^3^] refers to the total spatial volume of the resulting tracts, calculated as the number of voxels multiplied by voxel size. CC = corpus callosum, ILF = inferior longitudinal fasciculus, IFOF = inferior fronto-occipital fasciculus, SLF = superior longitudinal fasciculus. The effect size threshold corresponds to the Spearman correlation coefficient (ρ).

**Table 4:** Significant longitudinal changes (scan 2–scan 1) in QA and FA in the intervention group relative to the treatment-as-usual group.

Effect Size	QA ↑	QA ↓	FA ↑	FA ↓
0.25	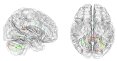	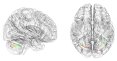	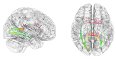	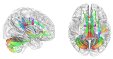
0.30	**—**	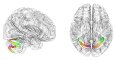	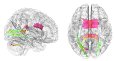	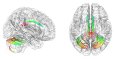
0.35	**—**	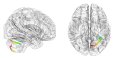	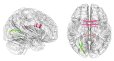	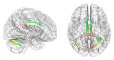
0.40	**—**	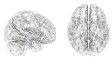	**—**	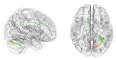

Note: QA = quantitative anisotropy; FA = fractional anisotropy; ↑ increase; ↓ decrease. Directional color-coding is used for visualization.

## Data Availability

The data from this study are openly available on the Austrian NeuroCloud at https://doi.org/10.60817/Z1GD-0Z95.

## Supplementary Materials

The following supporting information can be downloaded at: https://www.mdpi.com/article/10.3390/brainsci16101050/s1. Supplementary Material S1 contains a description of the dance intervention, informed by the TIDieR checklist [49] and recommendations for reporting dance research [50]. Supplementary Material S2 contains the baseline differences in diffusion metrics between the groups. Table S1: Baseline differences in QA and FA between the intervention group and the treatment-as-usual group, volume [mm^3^].
