# Supplementary material for: Changes in Diffusion MRI-Based White Matter Metrics After a Dance Intervention in Individuals with Substance Use Disorders—A Pilot Study
Source: Brain Sci. 2026 Sep 29;16(10):1050. doi: 10.3390/brainsci16101050 (PMC13702424; doi:10.3390/brainsci16101050)
Supplement: Supplementary file 1 [file brainsci-16-01050-s001.zip › Supplementary Material S1_Intervention.pdf]

Supplementary Material

Description of the dance intervention informed by the TIDieR checklist (Hoffmann et al., 2014) and recommendations for reporting dance research (Barnstaple et al., 2024)

**1. Why**	• Provided in the primary paper

**2. Location**	• The intervention took place in the rehabilitation facility, easily accessible for participants<br>• Large event space with wooden floor, natural lighting, no mirrors

**3. What & how**	
3.1. Dance Styles	• Mixed-genre dancing (including contemporary dance, creative dance activities, street dance, and classical ballet elements)
3.1.1. Rationale	• Dance styles were chosen due to their structure, principles, use of space, social factors, and movement qualities, as well as based on the research team's experience and expertise<br>• Accessible styles combining rhythm, expression, and variety<br>• Street dance elements (hip-hop-inspired) included to enhance musicality and motivation<br>• Creative dance activities were used to promote enjoyment and play<br>• Classical Ballet components integrated into warm-ups for technical input<br>• Contemporary dance was selected due to its versatility, focus on body awareness, breath control, and collaborative partner and team work, providing both expressive and physical challenges
3.1.2. Culture and Technique	• Street dance movements were used without replicating their traditional settings, such as cyphers, the elements were designed as hip-hop-inspired rather than formal hip-hop instruction, acknowledging that authentic street dance encompasses broader cultural and social elements<br>• Contemporary dance was informed by principles like counterbalance, use of breath, artistic exploration, and contraction/release
3.1.3. Incorporation	• Technical elements from ballet (e.g., tendu) and street dance styles were integrated into the warm-up<br>• Creative dance activities were included to encourage joy in movement beyond technical focus<br>• Contemporary dance elements were integrated throughout the class, including floorwork, partner work, and group work<br>• Instruction was primarily in German (occasional use of common French or English dance terms)<br>• Session 1-3 = choreography A; commercial, hip-hop-inspired<br>• Session 4-6 = choreography B; contemporary dance

3.2. Teaching Concept	• Participants were addressed as "dancers" or "class participants" (not "patients") and spoken to using the informal "Du" form (German)<br>• The intervention followed a co-teaching model, with instructors rotating roles three times per session between teaching and observing/providing support<br>• A variety of teaching methods were employed, including verbal guidance, mirroring, demonstration, and alternating between internal and external focus (for details on specific methods and techniques, see "Exercises" section)<br>• The teaching concept included a variety of methods and techniques, such as verbal instruction, mirroring, demonstration, imitation, alternation between internal and external focus, and the use of task-based as well as student-centered strategies
3.3. Class Structure	• Classes consisted of a warm-up, technique exercises, partner/group work, creative movement activities, choreography, and a cool down<br>• Warm-up & Dance Technique (20-25 minutes)<br>• Partner or Group Work; Creative Dance Activities (20-25 minutes)<br>• Choreography Practice (25-30 minutes)<br>• Cool-down (5-10 minutes)

**4. Class Details***	Goals	Methods & Techniques	Exercises
4.1. Warm Up *(Same structure; new focus each session)*	Activation and preparation	Demonstration & mirroring (frontal); free use of space in different directions; follow-along	Moving each part of the body with closed eyes; walking and running in different directions; spinal roll down/up; joint mobilization; movement flow (e.g., arms up in the air, arms down, roll down, plank position, roll up, repeat); awareness exercise: walking through the room, stopping, closing eyes, and pointing in the direction of something specific, such as "the person with red socks"
4.2. Dance Technique *(Repetition; build-up; new movements each session)*	Alignment and posture; control; balance; coordination; musicality	Demonstration & mirroring; frontal & across the room; use of levels, including the floor	Basic ballet movements (plié, tendu, relevé); contemporary floorwork (e.g., X rolls, monkey jump, push and pull, spins, transitions to and from the floor); basic street dance movements (e.g., bounce, criss cross)
4.3. Partner & Group Work *(New tasks in each session)*	Communication; trust; support; creativity; connection	Demonstration & observation; guided exploration	Eye contact and non-verbal connection ("Follow the gaze"); mirroring; weight sharing and transfer; impulse and response through touch; counterbalance exercises (standing back-to-back "glued" or holding hands and leaning backward); embodied group

			images; trust falls and catching exercises; lead and follow through touch;
4.4. Creative Movement Activities *(New tasks in each session)*	Exploration; body awareness; creativity; joy; motivation; playfulness; creation of movement	Task-based exploration; free use of space	Guided improvisation exercises (use of negative space; expression of a specific emotion through movement); "magic moment" during which half of the group moved gently with small movements and closed eyes to calming music, while the other half had the opportunity to observe<br><br>Dance Making 1: "I'm going on a picnic" game in a circle with dance movements; Dance Making 2: Story writing in groups and communicating through dance
4.5. Choreography *(Repetition; new elements added to the choreography in each session)*	Storytelling; synchronization; expression; timing; dynamics; flow	Demonstration & mirroring; call & response; phrase teaching; front-faced choreographies with limited use of space, except for levels and steps in each direction; structured in 8-counts	Sessions 1–3: commercial, hip-hop-inspired choreography<br>Sessions 4–6: contemporary dance choreography
4.6. Cool Down *(New exercises each session)*	Relaxation; release	Verbal guidance & instruction	Relaxation exercises including breathwork (waving the breath through chest, ribs, and stomach; box breathing); progressive muscle relaxation; muscle tension release exercise in groups (lying on the floor, releasing tension while the rest of the group gently moves limbs and head); "shaking it off" (dancing as "wildly" as participants choose, ending the song with a calm stillness on the floor).

*We aimed to include as much relevant information as possible; However, describing every single movement in detail would exceed the scope of this supplementary material. Improvised sections were not documented in order to maintain the flow of the sessions. For further exchange or clarification, please contact the author directly.

5. **Who**	Gender	Age	Experience	Background
5.1. Instructor A *(Involved in dance curriculum planning,*	female	26	Teaching experience in dance for health and community dance projects	Sports and Exercise Sciences; Dance Performance; Dance Teaching; Classical Ballet; Contemporary Dance; Street Dance Styles; Jazz Dance

delivery, and research)				
5.2. Instructor B *(Involved in dance curriculum planning, delivery, and research)*	female	28	Working experience in social and clinical contexts, including addiction	Psychology; Dance Performance; Classical Ballet; Contemporary Dance; Street Dance Styles; Jazz Dance;

**6. How much**	
6.1. Project Duration	• 3-week dance intervention
6.2. Session Duration	• 90 minutes
6.3. Frequency	• 2 sessions per week
6.4. Total of Sessions	• 6 sessions
6.5. Intensity	No physiological data (e.g., heart rate or exertion levels) were collected during the dance sessions

**7. Tailoring**	
7.1. Clothing	• Dancing in comfortable clothing and socks; option to keep shoes on; individual choice
7.2. Sharing	To accommodate individual comfort levels and support autonomy, participants were invited to engage in sharing activities. Three such situations were integrated into the intervention:<br><br>• Participants danced stories created by other groups to express those narratives through movement; all groups participated actively while others observed<br>• Participants were invited to present a short choreographic phrase in front of the group; participation was voluntary, and not all individuals chose to join<br>• In the “Magic Moment” exercise, participation was optional, but all participants chose to take part
7.3. Movement	• Movements and combinations were adapted to individual needs and abilities (Options included omitting arm movements, focusing on leg movements first, and offering slower or simplified versions of exercises)<br>• Suitable for adults with no dance experience
7.4. Speed and Tempo	• The pace of instruction was adjusted to match participants’ needs<br>• During across-the-floor exercises, particularly those involving floorwork, participants were encouraged to choose their own tempo
7.5. Improvisation & Creative Tasks	• Improvisational and creative tasks were intentionally designed to allow for individual choice and personal expression<br>• Participants were encouraged to explore and decide on their own tempo, movement dynamics, spatial use, direction, narrative, and forms of interaction
7.6. Music	• Music selection was adapted based on participants’ preferences to support motivation and engagement<br>• Before the first session, participants were asked about their musical tastes to avoid using genres they disliked

	• If a particular song was well received by the group or requested by a participant, it was used in future sessions to maintain high motivation and a sense of familiarity
7.7. Time of the day	• Sessions were scheduled in late afternoon to accommodate and align with the participants' rehabilitation schedule

**8. Modifications**	• Scheduled minutes were not always strictly followed when participant engagement or focus on a particular aspect required more time

**9. How well**	
9.1. Planned	• Dance Intervention Protocol, Pre-curated Music, Teaching Concept, Attendance List
9.2. Actual	• No changes were made to the protocol or music playlist<br>• Song selection varied by tempo and session needs<br>• Instructor A missed one session due to illness
9.3. Participant Feedback	• One participant dropped out after expressing dislike for dancing<br>• Feedback was informal, with space for questions after cool-down exercises<br>• Control group was also offered dance sessions, and some intervention participants requested to join again, indicating enjoyment<br>• Participants asked for song names, supporting the choice of music
9.4. Ecological Validity	• The intervention closely resembled a standard dance class for the target population, with participants aware that they were taking part in a study and that the instructors were part of the research team

**10. Safety**	
10.1. Environment	• Spacious event location with natural lighting and wooden floors<br>• Friendly, respectful communication to create a safe environment<br>• Easy access as part of the rehabilitation facility
10.2. Well-Being	• Check-in circle at the start and end of each class<br>• Invitation for individual choices and water breaks as needed<br>• Consent check for touch at the beginning (participants closed eyes and used a signal if they preferred no touch that day)
10.3. Support	• Co-teaching concept allowed the "observer" to participate alongside the group or provide individual support while the main instructor focused on other participants

**11. Music**	
11.1. Choreography Songs	• Choreography 1: “Stressed Out” by Twenty-One Pilots; Alternative hip hop, indie pop, and rock elements<br>• Choreography 2: “Young Blood” by Noah Kahan; Indie-folk with subtle pop influences
11.2. Choice of Music	• Music was selected by the two instructors to match the energy, structure, and goals of each task<br>• Participants’ music preferences were collected beforehand to avoid disliked music genres

	• Playlist included hip hop, indie, rock, electronic, and instrumental tracks to balance movement support and motivation<br>• Some songs were pre-assigned to exercises; others were chosen spontaneously during sessions<br>• Story Writing and Dance Making: Participants selected their own music to support creativity and personal connection
11.3. Use of Music	• Music was used in most exercises, with some moments intentionally done in silence<br>• Energizing tracks supported warm-ups; calm, atmospheric music used for focused or partner-based work<br>• Songs selected from a pre-curated playlist, with final choices made spontaneously to match session dynamics<br>• Choreography A (commercial, hip-hop-inspired): emphasized rhythm and groove<br>• Choreography B (contemporary dance): focused on expression and breath
11.4. Silence	• Check-in Circle: The session began without music<br>• Instructions were given without music (exception: warm-up; follow-along approach)<br>• Choreography Learning: Choreography was first taught in silence to allow participants to concentrate on movements before music was added<br>• Silent Exercises: Certain exercises, such as group story-writing, were carried out without music
11.5. Volume	• Portable speakers used throughout, with volume adjusted per exercise<br>• Lower volume for verbal instructions, explanations, and partner work<br>• Higher volume for movement phases like warm-ups and choreography<br>• Soft background music during cool-down to promote relaxation and body awareness

**12. Social**	
12.1. Eye Contact	• Follow the Gaze: Participants worked in pairs, maintaining eye contact while moving through the space<br>• Mirroring: Participants stood face-to-face and mirrored each other's movements, maintaining eye contact<br>• For certain exercises (e.g., "I'm going on a picnic"), the group was arranged in a circle, allowing participants to face each other directly<br>• During partner work, eye contact naturally occurred<br>• In front-facing exercises, instructors often looked toward the participants (since no mirrors were available)
12.2. Touch	• Leading and Following: One participant placed their hand gently on the back of another and guided them through the space<br>• Trust Fall: Participants practiced controlled backward falls, relying on the group to catch and support them, fostering trust and responsiveness within the group dynamic<br>• Impulse Work: Light impulses were initiated at various body parts (e.g., shoulder, hip, hand), encouraging participants to respond intuitively with movement

	• Counterbalance: Partner work included counterbalance exercises, such as leaning away from each other while maintaining connection (e.g., back-to-back or holding hands)<br>• Passive Movement (Cool-down): In a lying position on the floor, participants fully released muscular tension (group members gently moved their arms and legs, allowing for a passive, calming experience of being moved)<br>• No physical contact between instructors and participants; Exceptions: (1) instructors joined partner exercises to balance groups, (2) direct contact was used for clarity in partner exercise demonstrations
12.3. Choice of Partner/Group	• Participants were randomly grouped or paired based on their initial positioning in the room
12.4. Familiarity	• Participants were familiar with each other, as they were all undergoing rehabilitation at the same institution

References

Barnstaple, R. E., Laurita-Spanglet, J., Fanning, J., Soriano, C., & Hugenschmidt, C. E. (2024). This time with feeling: Recommendations for full-bodied reporting of research on dance. *Frontiers in Cognition, 3*, 1385087. https://doi.org/10.3389/fcogn.2024.1385087

Hoffmann, T., Glasziou, P., Boutron, I., Milne, R., Perera, R., Moher, D., Altman, D., Barbour, V., Macdonald, H., Johnston, M., Lamb, S., Dixon-Woods, M., McCulloch, P., Wyatt, J., Chan, A., & Michie, S. (2014). Better reporting of interventions: Template for Intervention Description and Replication (TIDieR) checklist and guide. *BMJ, 348*. https://doi.org/10.1136/bmj.g1687
